# CAREGIVER NETWORK BURDENS FOR OLDER ADULTS WITH DEMENTIA RISK: FINDINGS FROM NHATS AND NSOC DATA

**DOI:** 10.1093/geroni/igae098.0326

**Published:** 2024-12-31

**Authors:** Joseph Svec, Natasha Nemmers, JeongEun Lee

**Affiliations:** St. Joseph’s University, Patchogue, New York, United States; Wayne State University, Detroit, Michigan, United States; Iowa State University, Ames, Iowa, United States

## Abstract

A growing body of research indicates that caregiver networks constitute an important resource for older adult well-being as well as the adult caregivers themselves. Among dementia patients, caregiver networks often face heightened strain and burdens which correspond with poor overall health of all caregivers. However, the condition of a caregiver network is also shown in the literature to have a direct impact on the quality of care that is provided. This study examines the bi-directional nature of caregiver and care-recipient outcomes by examining the extent to which lagged measures of caregiver network burden corresponds with current dementia risk for older adults. We utilize data from the National Health and Aging Studies (NHATS) and the National Study of Caregiving (NSOC) to test associations of lagged caregiver burden within networks (e.g., financial, emotional, or physical difficulties) with care recipient dementia classifications (e.g. no dementia, possible dementia, probable dementia). Results from multinomial logistic regressions of four NSOC-NHATS data waves (N = 1,343) indicate that the odds of older adult possible dementia increase by 1% (OR = 1.01, p < 0.01) with each additional percentage increase in emotional difficulty shares within networks from the previous wave. Moreover, the odds of probable dementia increase by 2% (OR = 1.02, p < 0.01) with regards to both emotional and physical difficulty shares within caregiver networks. These findings suggest the importance of developing interventions that address diverse burdens by strengthening care coordination, communication, and support across network members to improve the cognitive health outcomes of older adults.

